# Offenders become the victim in virtual reality: impact of changing perspective in domestic violence

**DOI:** 10.1038/s41598-018-19987-7

**Published:** 2018-02-09

**Authors:** S. Seinfeld, J. Arroyo-Palacios, G. Iruretagoyena, R. Hortensius, L. E. Zapata, D. Borland, B. de Gelder, M. Slater, M. V. Sanchez-Vives

**Affiliations:** 1 Institut d’investigacions Biomèdiques August Pi i Sunyer, Systems Neuroscience, Rosselló 149-153, 08036 Barcelona, Spain; 2 Experimental Virtual Environments for Neuroscience and Technology (EVENT) Laboratory, Department of Clinical Psychology and Psychobiology, University of Barcelona, Passeig de la Valld’Hebron 171, 08035 Barcelona, Spain; 3 Brain and Emotion Laboratory, Department of Cognitive Neuroscience, Faculty of Psychology and Neuroscience, Maastricht University, Oxfordlaan 55, 6229 EV Maastricht, The Netherlands; 4 ICREA, Passeig Lluís Companys 23, 08010 Barcelona, Spain; 5 Present Address: Sony Interactive Entertainment, Research and Development, California, United States; 6 Present Address: Wales Institute for Cognitive Neuroscience, School of Psychology, Bangor University, Bangor, Wales United Kingdom; 7 Present Address: RENCI, The University of North Carolina at Chapel Hill, North Carolina, United States

## Abstract

The role of empathy and perspective-taking in preventing aggressive behaviors has been highlighted in several theoretical models. In this study, we used immersive virtual reality to induce a full body ownership illusion that allows offenders to be in the body of a victim of domestic abuse. A group of male domestic violence offenders and a control group without a history of violence experienced a virtual scene of abuse in first-person perspective. During the virtual encounter, the participants’ real bodies were replaced with a life-sized virtual female body that moved synchronously with their own real movements. Participants' emotion recognition skills were assessed before and after the virtual experience. Our results revealed that offenders have a significantly lower ability to recognize fear in female faces compared to controls, with a bias towards classifying fearful faces as happy. After being embodied in a female victim, offenders improved their ability to recognize fearful female faces and reduced their bias towards recognizing fearful faces as happy. For the first time, we demonstrate that changing the perspective of an aggressive population through immersive virtual reality can modify socio-perceptual processes such as emotion recognition, thought to underlie this specific form of aggressive behaviors.

## Introduction

Theoretical models of aggression have suggested that the perpetration of violent acts against others is linked to a lack of empathy or to a deficit in the ability of offenders to put themselves in the perspective of their victims^1^. In fact, some empirical studies have established a link between aggression and empathy^2,3^. For example, studies have found that offenders have difficulties in accurately recognizing emotions such as fear and anger, which has been hypothesized to hinder offenders’ compassionate responses^4^. However, other studies have failed to replicate these findings^5^. Discrepancies between studies might arise due to the multidimensional nature of empathy (ranging from emotion perception to cognitive perspective-taking), the differences in the methodologies used to assess empathy, the use of heterogeneous populations, and constraints found when trying to induce empathic responses in a laboratory setting^6^. In this sense, standard experimental studies aiming to understand core aspects of empathy often lack ecological validity due to ethical concerns. It is considered unethical to recreate in a laboratory a situation capable of inducing intense feelings of fear or distress in participants^7^. Additionally, research linking empathy and aggression has been frequently carried out in non-clinical populations, casting doubt on the transferability of these results to violent populations.

Here we introduce a new paradigm to study empathy and aggression in violent populations, which includes and goes beyond perspective-taking, since it allows participants to vividly experience a violent virtual situation from the perspective of the victim. This work is based on recent findings that have used immersive virtual reality (VR) to induce full body ownership illusions. In these studies, participants experience the perceptual illusion of ownership over a life-sized virtual body that visually substitutes their own body. Despite the fact that these virtual bodies may look drastically different to the participants’ own bodies in terms of size, height, skin tone, or age, individuals can still experience a strong subjective feeling of ownership^8,9^. Interestingly, such embodiment illusions can also influence social cognition by altering participants’ perceptions, attitudes and behaviors^10–13^. Furthermore, VR scenarios afford ecologically valid experimental setups while maintaining a high degree of experimental control. In these virtual scenarios, individuals have the illusion of being in a real environment and behave accordingly^14^. Therefore, VR provides a valuable tool to simulate and study violent behaviors without exposing participants to any real danger, hence overcoming the ethical issues that arise in non-virtual experiments^15^. This has been previously shown in several studies in which VR was used to assess sexually deviant behaviors of child molesters^16^, replicate Stanley Milgram’s obedience experiments^17^, and to study bystander responses to a violent incident to better understand helping and aggressive behaviors^18^.

**Figure 1:**
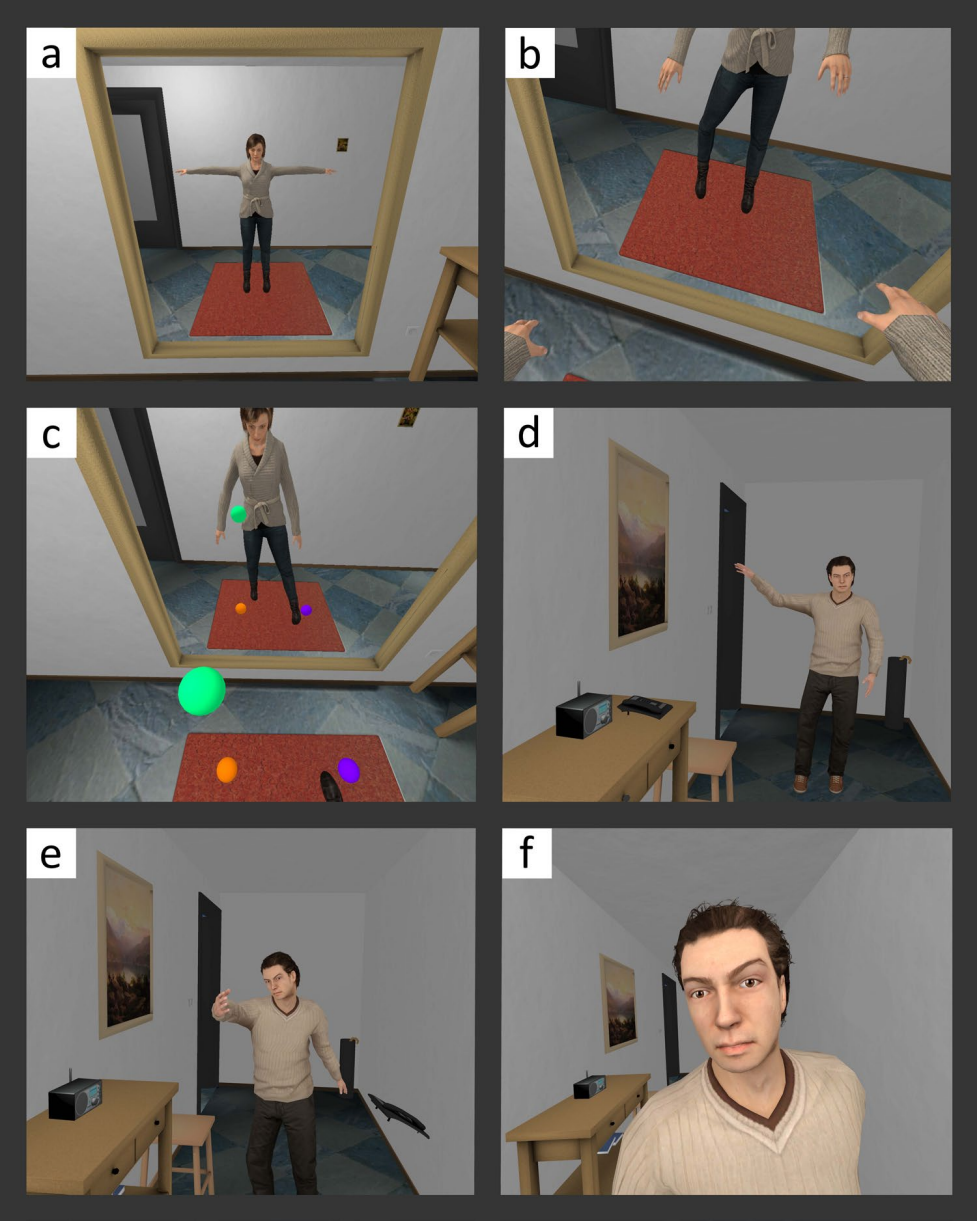
The immersive virtual reality scenario. (**a**) The participant looks at his female virtual body in the mirror. (**b**) The participant looks down towards his own body from a first-person perspective. (**c**) The participant touches the virtual balls. (**d**) The male virtual character enters the room and starts to verbally abuse the female virtual character. (**e**) The male virtual character throws a telephone on the floor. (**f**) The male virtual character invades the personal space of the participant.

The main goal of this study was to investigate some of the mechanisms of a type of violent behavior: domestic violence. Specifically, we have studied the impact of perspective taking (through virtual embodiment) and empathy on emotion recognition in participants including domestic violence offenders and controls. To this end, male participants entered a virtual environment where their body was substituted by that of a virtual female and they went through a process of “embodiment” (Fig. 1a–c). From this perspective, they saw a virtual male entering the scene and exhibiting abusive speech and gestures along with a progressive invasion of the victim’s (i.e., the participant’s) personal space (Fig. 1d–f; Video S1). The environment was interactive as the virtual abuser gazed towards the face of the participant shouting “Shut up!” if they spoke, or “Look at me!” if they looked away. We assessed emotion recognition skills before and after the VR experience, the degree of body ownership illusion^12^, and the subjective opinion regarding the virtual reality experience.

## Results

**Figure 2:**
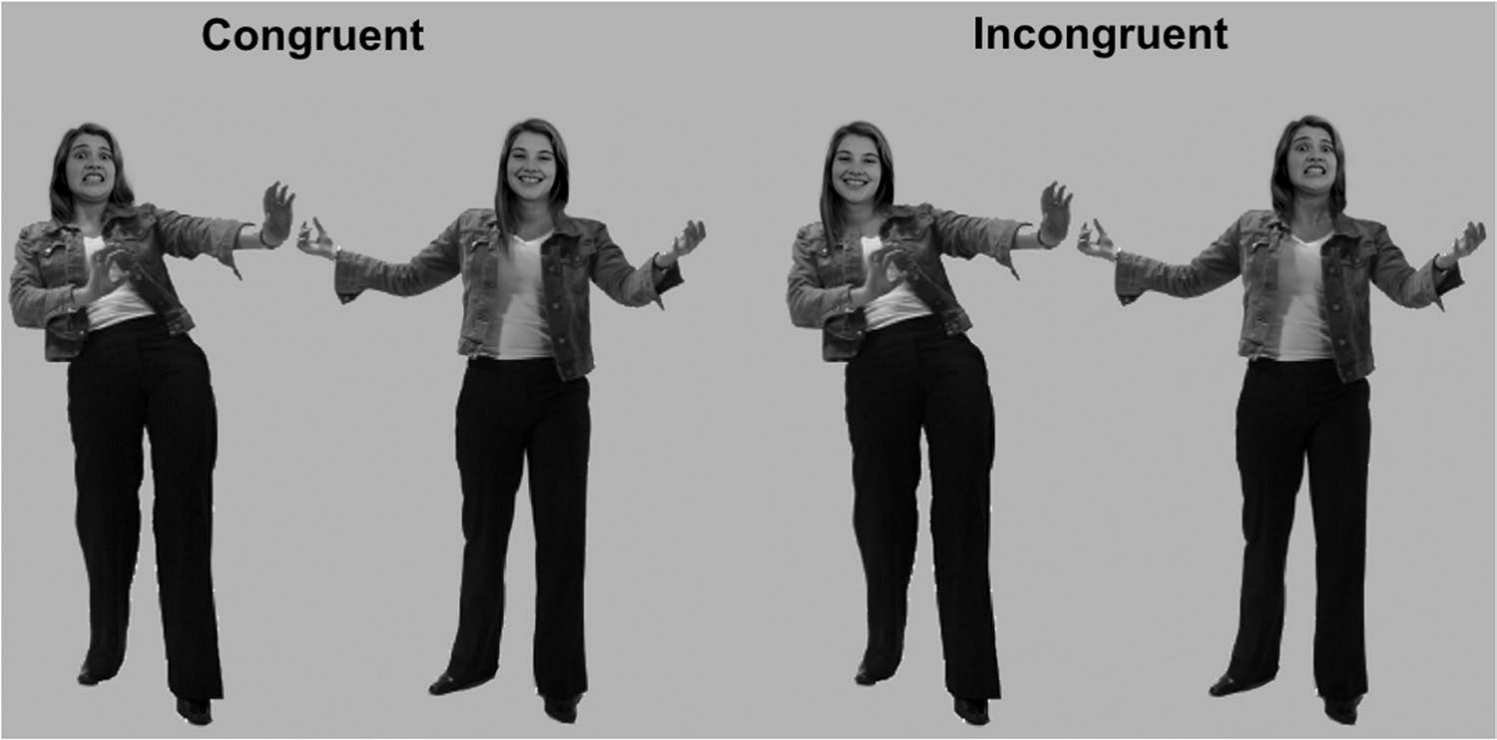
Face-body compound test to assess emotion recognition. Example of the emotionally congruent and incongruent face-body compounds of fearful and happy emotional expressions.

The experimental design was between-groups with one factor that we refer to as Condition, consisting of two groups of males: Offenders (n = 20) and Controls (n = 19; see Table S1 for demographic information). Participants completed the Face-Body Compound emotion recognition test before and after the VR exposure. In this test, faces expressing fear, anger or happiness were displayed for 100 ms on a computer screen. Each face was shown in isolation or on top of a body. The body expressed either the same emotional state as the face (congruent) or not (incongruent)^19,20^ (Fig. 2). Participants were given a forced-choice option (happiness vs. fear or happiness vs. anger) to categorize the facial expression of the different stimuli, and they were instructed to ignore the body. The test consisted of two independent blocks presented in a counterbalanced order. In one of the blocks, faces expressing either fear or happiness were displayed, and the other block followed the same methodology as the first one, but this time fearful faces were substituted by angry ones. Signal detection analysis was used to calculate the sensitivity index (*d′*) and the response criterion (*c*) of the recognition of angry and fearful facial expressions in male and female stimuli^21–23^. A higher value of *d′* means greater sensitivity for detecting angry or fearful faces compared with happy faces. The *c* criterion indicates the overall response bias. A negative *c* reflects a bias to respond with either angry or fearful, while a positive *c* indicates a bias to respond with happy to the emotional expressions. A *c* around zero suggests no bias in indicating the emotion of the presented expressions.

These scores resulted in 8 response variables as shown in Table 1. An additional response variable was a ‘Social Desirability Scale’ which measures the extent to which participants tend to respond truthfully or misrepresent themselves in order to put forward a positive self-representation.

**Table 1:** Response variables from the signal detection analysis included in the Bayesian Analysis.

Variable	Meaning
*d′*AngerMale	d′ score for anger recognition in male faces. Higher scores indicate more sensitivity to detect angry faces in male stimuli.
*d′*AngerFemale	Same for female faces.
*d′*FearMale	d′ score for fear recognition in male faces. Higher scores indicate more sensitivity to detect fearful faces in male stimuli.
*d*′FearFemale	Same for female faces.
*c*AngerMale	c-criterion for anger recognition in male faces. 0 = no bias, positive value = tendency to answer with happiness, negative value = tendency to answer with angry.
*c*AngerFemale	Same for female faces
*c*FearMale	c-criterion for fear recognition in male faces. 0 = no bias, positive value = tendency to answer with happiness, negative value = tendency to answer with fear.
*c*FearFemale	Same for female faces.

A Bayesian analysis was carried out that consisted of 9 simultaneous linear models^24,25^. Each model has as its response variables the post-VR exposure variables. The independent factor was the Condition, and the pre-VR exposure was an Offset variable. Details are given in the **Materials and Methods** section.

The equations model the difference between post-VR and pre-VR as a linear model of Condition (equivalent to ANOVA) such that a positive value of the coefficient of the independent factor (Condition) indicates that the difference between post-VR and pre-VR is greater for Offenders than Controls. There is a similar model for the pre-VR exposure variable, solely as a function of Condition, in order to check any differences in the response variables prior to the VR exposure.

Table 2 summarizes the results obtained and below we consider each in turn. It is important to note that this methodology specifies one overall model of the posterior distributions of all the parameters simultaneously.

**Table 2:** Posterior Probabilities that the Offenders and the Control groups differed in social desirability and in emotion recognition before (B) the immersive virtual reality (VR) experience and the change afterwards (post-pre).

Response Variable	Posterior Probability that Offenders > Controls on corresponding response variable	Interpretation
Social Desirability	0.98	Very strong evidence that Offenders exhibit greater Social Desirability than Controls.
B_ *d*′AngerMale	0.16	Some evidence that Controls better recognize male angry faces on a baseline level compared to the Offenders.
B_*d*′AngerFemale	0.53	No evidence either way.
B_*d*′FearMale	0.15	Some evidence that Controls better recognize male fearful faces on a baseline level compared to the Offenders.
B_*d*′FearFemale	0.03	Strong evidence that Controls better recognize female fearful faces on a baseline level compared to the Offenders.
B_*c*AngerMale	0.65	No evidence either way.
B_*c*AngerFemale	0.41	No evidence either way.
B_*c*FearMale	0.99	Overwhelming evidence that Offenders have a greater bias towards classifying male faces as happy rather than fearful on a baseline level when compared to the Controls.
B_*c*FearFemale	0.80	Good evidence that Offenders have a greater bias towards classifying female faces as happy rather than as fearful on a baseline level when compared to the Controls.
*d*′AngerMale	0.58	No evidence either way.
*d*′AngerFemale	0.10	Strong evidence that the VR experience produced a greater increase in the recognition of angry female faces in Controls than in Offenders.
*d*′FearMale	0.41	No evidence either way.
*d*′FearFemale	0.99	Overwhelming evidence that the VR experience produced a greater increase in the recognition of fearful female faces in Offenders than it did in Controls.
*c*AngerMale	0.51	No evidence either way.
*c*AngerFemale	0.74	Some evidence that the VR experience increased the tendency to classify female faces as expressing happiness to a greater extent in Offenders than it did in Controls.
*c*FearMale	0.04	Strong evidence that the VR experience increased the tendency to classify male faces as expressing fear to a greater extent in Offenders than it did in Controls.
*c*FearFemale	0.17	Good evidence that the VR experience increased the tendency to classify female faces as expressing fear to a greater extent in Offenders than it did in Controls.

Bayesian statistical model using Group as the independent variable. B, baseline (before VR); *c*, bias towards classifying faces as expressing a particular emotion; *d′*, sensitivity to recognize a particular facial expression.

### Sensitivity to recognize facial emotions (*d*′)

We first investigated the *baseline* (pre-VR) differences between domestic violence Offenders and Non-Offenders in their sensitivity (*d′*) to detect angry and fearful facial expressions against happy ones. The Control group recognized female fearful expressions more accurately (B_d′FearFemale, posterior probability = 0.97) than the Offenders group. They also recognized fearful (B_d′FearMale) and angry (B_ d′AngerMale) male facial expressions more accurately (both probabilities >0.84), but not angry female facial expressions (B_d′AngerFemale, posterior probability approximately 0.5) (see Tables 2 and 3).

**Table 3:** Means (Standard Errors) of Baseline (B) Sensitivity (d′) to recognize facial emotions and Baseline (B) Response Bias (*c*) towards classifying faces as depicting a particular emotion in domestic violence male offenders and non-offender male controls.

	Offenders	Controls
B_ *d*′AngerMale	2.42 (0.17)	2.62 (0.11)
B_*d*′AngerFemale	2.66 (0.18)	2.65 (0.12)
B_*d*′FearMale	2.48 (0.15)	2.61 (0.11)
B_*d*′FearFemale	2.44 (0.14)	2.73 (0.09)
B_*c*AngerMale	0.02 (0.04)	0.00 (0.04)
B_*c*AngerFemale	0.05 (0.04)	0.07 (0.04)
B_*c*FearMale	0.03 (0.06)	−0.13 (0.04)
B_*c*FearFemale	0.17 (0.07)	0.11 (0.04)

See Table 2 for statistical interpretations using a Bayesian model.

**Figure 3:**
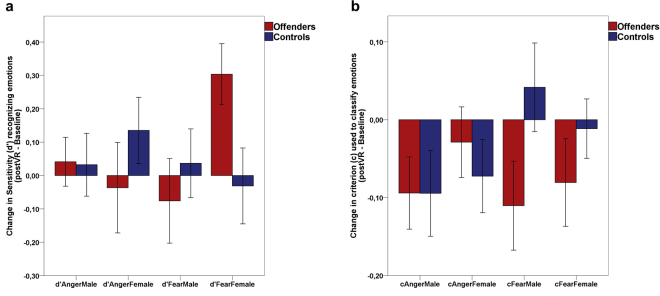
Change in sensitivity d′ (facility) to recognize facial emotions and in bias (c) towards classifying faces as depicting a particular facial emotion after the immersive virtual reality experience in domestic violence male offenders and non-offender male controls. (**a**) Means and standards errors of the differences (post-VR minus baseline) in sensitivity index d-prime (d′) scores obtained in the Face Body Compound Test, a higher value indicating a higher increase. (**b**) Means and standard errors of the differences in bias (c) in the Face-Body Compound test, where a c value of 0 indicates no change; a positive value of c indicates an increase in bias towards classifying faces as happy, and a negative c value shows an increase in bias towards classifying faces as angry or fearful after the VR experience. See Table 2 for statistical interpretations using a Bayesian model.

After the VR exposure, the Offenders group substantially improved their recognition of fearful female facial expressions compared to the Control group (d′FearFemale, posterior probability = 0.99). However, the Offenders remained worse than the Control group in recognizing angry female faces (d′AngerFemale, posterior probability = 0.90). There were no evident differences in the recognition of angry and fearful male expressions after the VR experience in either group (see Fig. 3a and Table 2). This reveals that male domestic violence Offenders have difficulties recognizing female fearful facial expressions compared to male Controls. However, this deficit improved after the VR exposure in Offenders, while the detection of fear in female faces in the Control group remained constant.

### Bias towards classifying faces as depicting a particular emotion (c)

Baseline, pre-VR, showed a bias towards classifying fearful female faces as happy in the Offenders with respect to the Control group (B_cFearFemale, posterior probability = 0.80). Moreover, Offenders have a greater bias towards classifying fearful male faces as happy compared to Controls (posterior probability = 0.99). However, there does not seem to be a difference in the bias (*c*) used to classify angry or happy stimuli between groups (see Tables 2 and 3).

Regarding the impact of the VR experience on the bias (*c*), our results suggest that the bias of the Offenders towards incorrectly classifying fearful male faces as expressing happiness was reduced after the VR compared to the Control group (posterior probability = 0.96). This was also found for fearful female faces (posterior probability = 0.83). There was no evident difference between groups in the change in bias after the VR experience towards classifying angry faces as angry or happy (see Fig. 3b and Tables 1 and 2).

### Social desirability, empathy, and subjective perception of the VR

A questionnaire on social desirability revealed that the Offenders group (Mean = 20.30, Standard Error = 0.88) had higher scores than Controls (Mean = 17.42, Standard Error = 1.09). This suggests a tendency of Offenders to answer in a manner that would be viewed favorably by others, which calls into question the reliability of their self-reported questionnaires in general. The posterior probability that Offenders have higher scores than Controls on the Social Desirability Scale is 0.98 (Table 2). These results are in line with those of Andrews & Meyer^26^, who found that a large sample of males sentenced for domestic violence had a mean score of 18.77 (SD = 5.97) on the social desirability scale, and that such scores were significantly higher than those of male Non-Offenders. Therefore, following standard strategies used to deal with socially desirable responses^27^, the Interpersonal Reactivity Index^28,29^ and VR questionnaires data were not further analyzed, but descriptive results are presented in Supplementary Information (SI). The scores given by the Offenders cannot be regarded as reliable. This unreliability does not apply to the implicit measures such as the emotion recognition test, in which participants are presented with stimuli very briefly (100 ms) and have to give a very fast response that cannot therefore be based on a conscious decision.

## Discussion

The main goal of this study was to investigate the impact of perspective taking during violent behavior and the empathy that it engenders on emotion recognition in participants including domestic offenders and controls. A VR paradigm for embodiment of males in a female virtual body was carried out (Fig. 1a–c)^30,31^ for participants to experience the virtual scene as if they were the target of domestic violence. Our findings indicate that, at baseline levels, male domestic violence Offenders do not well-recognize fearful female facial expressions when compared to Control males (Non-Offenders). Furthermore, we found that domestic violence Offenders have a greater bias towards classifying both male and female fearful faces as happy, rather than fearful, compared to Non-Offenders. However, being embodied in a female victim who suffers verbal abuse and intimidation by a male character using VR resulted in an improvement of the ability of Offenders to recognize fear in female faces, and reduced their response bias towards wrongly attributing happy emotional states to fearful facial expressions, independently of gender. This effect occurred overall in Offenders and not in Controls. Our findings indicate that changing the perspective of an aggressive population by means of virtual embodiment in the victim impacts emotion recognition, which is a socio-perceptual process thought to underlie this specific form of aggressive behavior.

The deficit in fear recognition found in the Offenders group is in line with several studies that have shown a deficit in recognizing negative emotional states in violent populations^32^. Our data strongly support the notion that men who are violent towards their female partners are less able to recognize fear in female faces than non-violent men. Based on our findings, we hypothesize that this deficit can partially explain why domestic violence Offenders may behave abusively towards their female partners. This is in line with a previous study showing that the inability of these violent men to recognize fearful expressions in their wives or unfamiliar women correlates with intimate partner violence perpetration^33^.

Blair’s Violence Inhibition Mechanism model^34^ proposes that poor recognition of the distress or fear experienced by the other can prevent a compassionate and empathic response. Similarly, Simulation Theory suggests that deficits in the production of a specific emotion, and deficits in the recognition of that emotion, co-occur^35^. In fact, poor ability to recognize fear can also encompass problems in other socially relevant skills, such as theory of mind, and these abnormalities have been linked to impairments in the neural functioning of brain regions that underpin social information processing^36,37^. For example, structures such as the amygdala might be involved in these deficits, since it has been previously found that this region is crucial for the processing of threating signals under ambiguous or incongruent conditions^38,39^.

However, our study shows that this deficit can be reduced through the type of VR exposure that we employed here. Our results indicate that a virtual domestic abuse scene experienced from the perspective of a female victim can positively increase Offenders’ sensitivity to recognize fear in fearful female facial expressions, and reduce Offenders’ bias towards misclassifying facial expressions as expressing happiness rather than fear. We also found that there was an enhancement in the recognition of angry female faces in the Control group after the VR experience when compared to the Offenders group.

These results are in line with other studies that found that non-virtual body ownership illusions can specifically enhance the recognition of fear. Such an example is the “enfacement” illusion, in which the illusory feeling of ownership over a different face can be produced by seeing a face being touched while feeling touched synchronously and correspondingly on one’s own face. This illusion can facilitate the recognition of fearful expressions but not of other types of emotions^40^. It is hypothesized that shared multisensory experiences, such as embodiment over a different body, can facilitate simulation mechanisms in the somatosensory cortex that are specifically related to fear perception in others. In accordance with this, it has also been shown that the Visual Remapping of Touch effect (a phenomenon in which seeing a face being touched can enhance the observer’s accuracy in detecting tactile perception on their face) is modulated to a greater degree by fearful expressions than by other emotional expressions^41^. Additionally, individuals experiencing mirror-touch synesthesia, where subjects report feeling touch on their own body when they see someone else being touched, also better recognize emotional states presumably because of their ability to simulate others’ somatosensory experiences^42,43^.

Importantly, the enhancement of fear recognition after embodiment in a female victim only occurred in the domestic violence Offenders group, and not in the Control group. A possible explanation is that the emotion recognition test used in the present study was sensitive enough to detect changes in the Offender group, who had a deficit, and thus, larger room for improvement, but not in the Control group, who had better recognition of fearful faces to start with. This difference could also be explained in terms of how perception and cognition might be affected by previous personal history and exposure to violence^19^. Several studies have found that violent and non-violent populations differ in their perceptual-biases and allocation of attentional resources when confronted with threatening stimuli^44,45^. In this sense, it is possible that the subjective interpretations of the embodiment experience were different between the groups. In the case of offenders, the VR scene reverses their usual experienced perspective - they become a victim instead of a perpetrator. This could have resulted in a profound shift of perspective while still identifying with the male avatar, which only impacted their processing of fear related emotions. No such drastic change of perspective occurred in the control group participants, who might have allocated more attentional resources to the processing of threatening cues, which might in turn have enhanced their recognition of anger in female facial expressions after the IVR experience.

In the present study, we also found that Offenders seem to have lower sensitivity to accurately recognize anger in male facial stimuli compared with Controls, although we did not find this effect for female stimuli. Additionally, only small between-group differences were found in the bias towards classifying angry faces. These results are compatible with a study showing that generally-violent/antisocial men who also committed acts of intimate partner violence, taken from a community sample, tended to misidentify angry expressions as disgust or happiness^46^. However, the authors observed that such a pattern of anger recognition did not occur in family-only offenders or in borderline personality disorder offenders. Although we cannot directly compare our results with these findings since we did not follow the Holtzworth-Munroe & Stuart^47^ typology classification for male batterers, these studies highlight the need to distinguish between subtypes of intimate partner violence male abusers based on the severity of their violent behaviors. Future studies should aim to elucidate whether such patterns in facial emotion recognition ability can be replicated in a sample such as that of the present study, i.e. in men who are sentenced and enrolled in a rehabilitation program for domestic violence.

The results of the present study further support the notion that experiencing embodiment over a different body to one’s own can influence perceptions, attitudes and behaviors. This and other studies indicate that altering the perception of the self, from a purely bodily aspect, seems to also modify self-related socio-cognitive processes, which could be exploited in order to tackle real life problems^10^. For example, it has been demonstrated that embodiment of light-skinned people in a dark-skinned body can reduce implicit racial biases against that outgroup^11,48^, an effect that seems to be long-lasting^49^. Similarly, evidence also indicates that embodied perspective-taking in VR is a promising tool for facilitating the resolution of intrapersonal conflicts^13,50^. In the present study, for the first time, we provide evidence demonstrating that embodied perspective-taking through VR also has the potential to improve current rehabilitation programs for offenders by enhancing emotional recognition skills. Finally, our results also partially support the theoretical framework proposed by Yee and Bailenson^51^ (known as the Proteus effect), which states that a virtual self-representation can have a significant impact on behavior. These authors demonstrated that participants assigned to more attractive or taller avatars tended to exhibit more intimate and confident behaviors towards a confederate, compared to conditions in which participants were assigned to less attractive or shorter avatars. However, in the present study we did not test the effects of embodiment over a virtual female victim on subsequent inter-personal interactions nor the effect of self-stereotyping.

From a mechanistic point of view, we hypothesize that the novel multisensory experience of facing violence from a victim’s perspective can facilitate the identification of the potential distress and fear-related emotions experienced by victims of domestic violence. Such enhancement in emotion recognition could be at the basis of a behavioral change towards the victim through the reduction of future re-offenses, which is ultimately the desired outcome of the present study. Furthermore, improvement in emotion recognition could also be explained in terms of the impact that embodiment experiences can have on brain regions involved in affective face encoding, such as the somatosensory cortex, amygdala, cingulate cortex, ventromedial prefrontal cortex, and fusiform face area. The use of brain imaging techniques in future research will be crucial in order to understand the neural mechanisms implicated in the effects that embodiment can have on emotion recognition. Finally, the critical studies will be those which explore how these virtual reality experiences can impact recidivism of violent behavior.

Our findings also highlight the need to use measures that cannot be consciously manipulated when working with these types of populations, such as the emotion recognition test employed here or the recording of physiological reactions. In the present study we quantified the impact of the experience using an implicit measure that is independent of the questionnaire results and not affected by the increased social desirability detected in the Offenders group. Hence, the Face-Body Compound test was used, since a previous event-related potentials study showed that the congruency of such stimuli is processed within 86–128 ms, a timing that precludes conscious decision making^19,20^. Our study illustrates that implicit and explicit measures do not seamlessly map onto each other, as shown by the results obtained in the questionnaire-based responses that reflect the impact of enhanced social desirability amongst the Offenders. We found that domestic violence Offenders record higher scores in the Social Desirability Scale in comparison to Non-Offenders, suggesting that Offenders tend to respond in a way that they presume is socially desirable. This is in line with studies showing that violent populations try to give a compliant and positive image of themselves when enrolled in court rehabilitation programs^52^.

Furthermore, this study also contributes to the development of new strategies aimed to improve the efficacy of rehabilitation programs, which include empathy training or perspective-taking techniques based on watching movies, reading testimonies or carrying out role-playing activities. These activities aim to help Offenders better understand the feelings of their victims by putting them in the female’s shoes^53^. A major drawback of such techniques is that they are explicit in their message, providing verbal information rather than actual experience, and therefore may not impact the Offenders’ behaviors towards their victims. Although Offenders might obtain abstract knowledge that their behavior is wrong, this may not actually result in behavioral changes. Furthermore, the fact that such methodologies rely on the motivation and capacity of Offenders to take the perspective of the victim in a violent situation can be problematic for those with a lower capacity of imagination, or amongst those with a low level of motivation towards the treatment. The virtual embodiment paradigm used in this study overcomes such limitations by making the Offenders actually experience an abuse situation from the perspective of the victim, independently of motivational factors and of the participants’ facility to imagine hypothetical situations.

In this study we were interested in whether the shift in embodied perspective taking would result in observable changes when comparing the Offenders to the Controls. The methodology used in the present study was grounded on past research which showed that providing congruent multisensory information is crucial to induce a body ownership illusion^8^. However, in future work we aim to manipulate different parameters of the embodiment experience in order to explore the most relevant factors contributing to the changes in emotion recognition. For example, future studies should explore the impact on emotion recognition of conditions where participants are provided with incongruent multisensory information concerning their virtual body in order to test whether the observed effects are due to the body ownership illusions *per se*^54^. Furthermore, the role that the aggressive social interaction established by the male and female virtual characters and the stereotypes associated with the virtual female body played in the observed effects also remains to be answered^55^. In particular, it has been shown that domestic violence offenders frequently present stereotypes and cognitive distortions concerning females^56^. Future studies using a similar methodology should assess whether such changes in emotion recognition occur after experiencing embodiment over a male virtual character that is being abused or by simply experiencing embodiment over a virtual female body in a neutral scenario. Finally, future research should control for inter-individual differences through validated scales (e.g. personality traits, psychopathology, and dispositional emotional contagion) in order to analyze how such variables can influence the results.

In conclusion, accurately recognizing emotions is a key component of empathic responses and several studies have demonstrated that better recognition of fearful expressions predicts prosocial behavior^57^. The results of this study suggest that experiencing a virtual situation of domestic abuse from the perspective of the victim can result in an enhancement of fear recognition by the offenders. Although the implicit measures of emotion recognition are valid objective measures of the impact of this experience in violent perpetrators, this does not imply that all the effects are restricted to those currently measured. Our study is a first demonstration of the potential of immersive VR to induce positive changes in actual offenders. However, subjective, physiological, and explicit changes should be investigated in the future. Furthermore, the final objective should be to search for behavioral changes that contribute to reducing future re-offenses, thus contributing to the well-being of the victims, the offenders themselves, and society in general.

## Materials and Methods

### Sample

The *Offenders* group (N = 20) was recruited in collaboration with the Catalan Justice Department. This group consisted of men convicted by the Spanish legal system for an aggression against a woman and sentenced to attend a domestic violence intervention program.

The *Control* group (N = 19) consisted of men without a history of domestic violence. They were recruited via advertisement in the community and also from the maintenance staff of the University. The Control group matched the Offenders group in age, educational level, nationality and occupational status. The original sample had 44 participants, 23 Offenders and 21 Controls, but 3 were discarded as outliers and 2 due to missing critical information (for further demographic information and exclusion criteria, see SI Sample section).

This study followed ethical standards as per the Declaration of Helsinki and was approved by the Ethical Committee for Clinical Research at the Hospital Clinic of Barcelona. All included participants gave written informed consent (for more details see SI Further Ethical Considerations section).

### Measures

Emotion recognition was assessed before and after the VR experience with the Face-Body Compound test^19,58^. Face-body compound images were created by assembling emotionally congruent or incongruent body expressions with emotional faces of five male and five female actors (Fig. 2). These stimuli have been previously validated in a study that found that violent offenders have difficulties in processing emotional incongruence and have a possible bias towards perceiving emotional expressions as aggressive^19^.

We used an adaptation of the Face-Body compound test that consisted of two independent blocks presented in a counterbalanced order. In one of the blocks, faces expressing either fear or happiness were displayed in isolation or on top of a body that expressed the same emotional state (congruent) or not (incongruent). The other block followed the same methodology as the first one, but this time fearful emotional expressions were substituted by angry ones. When faces were displayed in isolation, no outlines of the bodies were visible and the space of the body was filled with a grey background color. However, the same size and position on the screen was preserved when compared to stimuli which included bodies. Subjects were given a forced double-choice option (happiness vs. fear or happiness vs. anger) to categorize the facial expression of the different stimuli and they were instructed to ignore the body. The stimuli were briefly presented (100 ms). After each response, a fixation cross appeared for 750 to 1250 ms. Each block of the test included 60 pictures (2 facial expressions * 3 body postures * 10 actors) that were presented twice in a random order. All participants completed this test before and immediately after the VR experience. This test was analyzed using a signal detection analysis (details in SI Signal Detection Analysis).

Furthermore, the Spanish version^59^ of the Social Desirability Scale was used to assess potential response bias in the other self-reported questionnaires since it measures a subject’s tendency to respond in a socially desirable way. The scale comprises 33 true-false items concerning everyday behaviors. Higher scores in this scale indicate a stronger tendency to portray a positive image of the self and a tendency to respond in what is presumed to be desired by society.

We also administered the Interpersonal Reactivity Index^60^ and a self-developed VR questionnaire, which were not included in the final study due to socially desirable responding (see more details on the analysis of these variables in SI IRI and VR questionnaire).

### Procedure and Virtual Scene

Upon arrival, all participants signed an informed consent form, and completed a demographics form, the IRI, and the social desirability scale. Subsequently, we administered the Face Body Compound Test (pre-VR). Participants sat 55 cm from the computer display.

Before the VR experience, participants were fitted with the head mounted display (HMD) and a pair of headphones. The VR scenario depicted a room with a long hallway where the participant’s own body was replaced with that of a virtual female. All participants saw the virtual female from a first-person perspective. The virtual body moved in real-time in accordance with the actual movements of the participants, producing visuomotor synchrony. The virtual female character stood at one end of the hallway facing a full-length mirror, which had a table with a radio and a phone on its side. Towards the other end of the hallway was an open door. A 1 × 1 m rug on the ground in front of the full-length mirror, below the virtual female, was used as a reference for the area in which the participant could stand and navigate.

First, we asked participants to become familiar with their virtual female body and the surroundings by looking around and moving their new virtual body (Fig. 1a). For this, the virtual female character faced the full-length mirror. We also asked them to look down towards their own body so that they could inspect their virtual body from a first-person perspective (Fig. 1b). Next, participants were asked to describe their new virtual body and the room. At this moment, participants heard some pre-recorded instructions while 4 virtual balls appeared in front of them. In order to enhance the body ownership illusion through visuomotor synchrony and agency, participants were instructed by audio to touch the virtual balls. Two virtual balls appeared high in the air to be touched with their hands and two at floor level to be touched with their feet. When a ball was touched it disappeared until all four were touched, then a new iteration of the exercise started where the balls appeared again in similar positions. Participants did three iterations of this exercise, which lasted approximately 2 min (Fig. 1c).

After doing the exercises, the virtual female body faced the far end of the hallway instead of the mirror. A male virtual character entered the room and began to verbally abuse the female virtual character following a pre-defined script (Fig. 1d). The dialogue of the abuser contained various insults against the female character. More specifically, the male character constantly derided the physical appearance and defenselessness of the female virtual character. He also made frequent references and threats regarding the fact that the female was financially dependent on him.

The male virtual character was programmed to constantly look into the eyes of the female character. The male character approached the female character and at a certain point he hit the telephone, which fell off the table in the direction of the virtual female body (Fig. 1f). This was done in order to include an act of physical violence. On three occasions during the verbal abuse, the male virtual character walked towards the female character, and by the end of the scene he was right in front of her (Fig. 1e). Additionally, if participants interrupted the monologue of the male virtual character at the start of the experiment, the experimenter could make the male character say “Shut up!”. The verbal abuse lasted for approximately 3 min 20 s (see more details in SI Procedure, Technical setup, and Movie S1).

Once the VR scene finished, all equipment was removed from the participants and they were asked to immediately complete the emotion recognition test (post-VR). Subsequently, participants filled in the post VR experience questionnaire, we carried out a semi-structured interview and we conducted the debriefing about the virtual experience.

### Statistical Analysis

For the *d′* and *c* variables shown in Table 1, each equation was of the form (1, 2):

1 $${\mu }_{post,i}={y}_{pre,i}+{\alpha }_{post}+{\beta }_{post}{C}_{i}$$

2 $${\mu }_{pre,i}={\alpha }_{pre}+{\beta }_{pre}{C}_{i}$$

3 $${y}_{post,i} \sim N({\mu }_{post,i},{\sigma }_{post}^{2})$$

4 $${y}_{pre,i} \sim N({\mu }_{pre,i},{\sigma }_{pre}^{2})$$

where ${y}_{post,i}$ and ${y}_{pre,i}$ are the *i*th observations of the variables in Table 1 as measured post and prior to the IVR exposure. ${y}_{post,i}$ is normally distributed with mean equal to offset ${y}_{pre,i}$ plus the linear model involving the unknown parameters ${\sigma }_{post}$ and ${\beta }_{post}$ where *C*_*i*_ is the Condition (Control = 0, Offender = 1) of the *i*th participant. The prior distributions of the *α′*s and *β′*s were taken as bivariate normal with mean vector (0,0), variances 1600 and covariance 160, thus highly non-informative. The prior distribution of the variances ${\sigma }^{2}$ were also taken as a highly non-informative Gamma distribution with parameters $r=\lambda =0.001$, where the probability density $f(y)\propto {\lambda }^{r}{y}^{r-1}exp(-\lambda y)$.

For the Social Desirability scale the same model was used, except that this was only measured prior to the VR exposure so that there is no offset term in the equation.

Analysis was carried out using the JAGS system^25^, together with MATLAB via MATJAGS (http://psiexp.ss.uci.edu/research/programs_data/jags/), and some graphs were produced using Stata 14 (see SI Bayesian Analysis).

The datasets generated during and/or analysed during the current study are available from the corresponding author on reasonable request.

## Electronic supplementary material

Supplementary Information

Supplementary movie
