# Supplementary material for: Offenders become the victim in virtual reality: impact of changing perspective in domestic violence
Source: Sci Rep. 2018 Feb 9;8:2692. doi: 10.1038/s41598-018-19987-7 (PMC5807352; doi:10.1038/s41598-018-19987-7)
Supplement: Supplementary file 1 — Supplementary Information [file 41598_2018_19987_MOESM1_ESM.pdf]

Offenders become the victim in virtual reality: impact of changing perspective in domestic violence

S. Seinfeld, J. Arroyo-Palacios, G. Iruretagoyena, R. Hortenisus, L. Zapata, D. Borland, B. de Gelder, M. Slater, and M. V. Sanchez-Vives

Supplementary Information

Materials and Methods

Sample

The exclusion criteria for the Offenders group were: not authorizing the anonymous use of their data to evaluate the VR application, having a low level of Spanish comprehension or suffering from epilepsy. Hence, although originally 37 offenders went through the VR experience, 17 were excluded, leaving a final sample size of N=20. Specifically, 6 participants did not authorize the anonymous use of their data for this study, 4 were non-native Spanish speakers, 2 were illiterate, critical information was missing for 2 participants, and 1 additional participant was excluded from the analysis because he showed up as an outlier in the data several times (more than two standard deviations from the mean).

The Control group was recruited by asking individuals interested in participating in the study to fill in a form with basic demographic information (age, nationality, education, marital status, history of psychiatric issues, medication intake, work status, existence of a criminal record) where they were also asked to judge the quality of their relationships with their family, intimate partner and friends on a rating scale with the options: very bad, bad, normal, good, and very good. The exclusion criteria for the Control group were the same as for the Offenders group as mentioned above, but also included: intake of psychotropic drugs, having a criminal record, being familiar with domestic violence as an offender or a victim, judging their close relationships with their intimate partner or family as bad or very bad, and having a history of psychiatric illness. Out of the original 24 participants, 2 subjects were excluded because they reported witnessing domestic violence during their childhood and another subject reported identifying with the violent behavior of the male virtual character. Two

additional participants were excluded from the analysis because they showed up as outliers in the data several times. Control participants were paid 15 euros for their participation.

The specific demographic characteristics of the Offenders and Control group can be seen in **Table S1**.

Table S1. Demographic information of the Offenders and Control groups.

	**Offenders group**	**Control group**
**Nationalities**	17 from Spain, 3 outside of Spain	18 from Spain, 1 from Chile
**Mean Age (SD, range)**	38.75 (8.52), 22-61	35.95 (10.63), 21-57
**Education**	5 finished primary school<br>7 finished high school<br>6 finished vocational training<br>2 finished a university degree	4 finished primary school<br>6 finished high school<br>7 finished vocational training<br>2 finished a university degree
**Work (yes, no)**	12, 8	15, 5
**Marital Status**	8 single<br>3 married or in a relationship<br>9 divorced	10 single<br>8 married or in a relationship
**Children (yes, no)**	15, 5	3, 16
**Medication**	3 on psychoactive medication	None on medication

Further Ethical Considerations

Concerning the content of the scene, participants were naïve regarding the type of virtual body they would have during the scene, or to the fact that we were studying how such experiences can impact emotion recognition. However, we informed the control group that the scene could contain offensive/disturbing images. More specifically, we included the following phrase in the information given to participants and in the consent form: “This virtual reality session may contain offensive or

disturbing images. You have the right to withdraw from the session at any moment, by indicating it to the researcher who will be with you throughout the whole session."

Furthermore, the offenders group was also informed beforehand that similarly to the exercises carried out in their own therapy and rehabilitation sessions based on watching movies or reading testimonies, in the virtual reality experience they would experience a scene related to the main topic of their rehabilitation course which was domestic violence.

Participants were informed that they could choose to discontinue the experience and withdraw of the study at any time by just removing the HMD or telling the experimenter. Moreover, at the end of the study, both groups were debriefed about the purpose of the experiment and checked through a semi-structured interview for any side-effects.

Taking into account further ethical considerations, a follow-up questionnaire was emailed after two weeks to the Control participants in order to check whether they had experienced any side effects of having been involved in the VR scenario. More specifically, the questionnaire assessed whether participants had any positive, negative, or strange feelings and thoughts after participating in the study. This same follow-up procedure was conducted with the Offender population by the therapists of the treatment program who carried out weekly treatment sessions. No specific negative consequences of the VR application were reported in any case.

Follow-up questionnaire (Controls)
English version

1) How much have you thought about this experiment in which you participated you're your answer is "yes", please state what type of thoughts you had.

2) To what extent did your experience of this scenario generate any negative thoughts or feelings (please describe)?

3) To what extent did your experience of this scenario generate any positive thoughts or feelings (please describe)?

4) Have you had any strange or unusual or unexpected thoughts or feelings in relation to your experience (Please describe if 'yes')?

5) Would you be prepared to be involved in a similar experiment again?

In case that you want us to contact you to talk about any unresolved feelings produced by your experience in this study, please give your name and email. If you do not want us to contact you about your experience please just write "NO".

Procedure

Men in the Offenders group were informed by their therapist prior to their participation in the study that one of the treatment sessions consisted of going through a VR simulation that would be discussed in subsequent group sessions.

The experiments with offenders were carried out in a consultation room of the Justice Department of Catalonia, whereas the experiments with the control men were conducted at the University of Barcelona. Although the location where the experiments took place was different for offenders and controls, the experimental setup was kept as similar as possible for both groups (i.e. the same equipment was used, same testing order and same experimenter).

On their arrival, offenders were informed verbally and with an information sheet that they would experience a VR scenario in which they would have to carry out some small movements with their virtual body in order to get familiarized with it. Furthermore, they were also informed that their main task would involve observing the different events happening in the virtual scene and that they would be free to interact by talking or moving if they wished to. However, no reference was made to the actual content of the scene or to the type of virtual body they would have. The information sheet also described the potential risks of VR use (e.g. dizziness and flashbacks). It was indicated that if at any time they felt dizzy or wanted to withdraw from the experience, they should report it to the experimenter. The experimenter was a qualified clinical psychologist who ensured that any participant who became distressed would receive appropriate support. Offenders were informed that all the information collected during the study was completely anonymous. However, before starting the study they were asked to sign a consent form if they were willing to authorize the use of the data

collected through questionnaires, computer tests, and interviews, to evaluate the effect of the VR session on the treatment.

In the case of the Control group the same procedure was followed.

All participants were informed that they were free to withdraw from the study at any moment without giving explanations to the experimenter and they were asked to sign a consent form if they were willing to participate.

Technical setup

The animation of the virtual male abuser character was created by recording the real-time voice, face and body movements of a professional actor. The abuser's dialog was inspired by prototypical gender violence situations described in court sentences and taking into account the suggestions of professional clinical psychologists. A pilot study was carried out in order to validate that participants judged as threatening several aspects of the scene such as the language used by the male character, throwing a telephone to the floor or invading participants' personal space.

The actor's facial movements were captured using a Primesense 1.09 Carmine depth camera (Primesense, Tel Aviv, Israel) and the data were interpreted using the facial motion capture software Faceshift (Faceshift, Zurich, Switzerland). To record the rest of the body, the actor wore a motion-capture suit with 37 reflective markers. The actor's performance was recorded using an OptiTrack (NaturalPoint, Oregon, United States) motion capture system with 12 cameras. Both facial and body movements were integrated, fine-tuned and adapted to the target character using MotionBuilder 2014 (Autodesk, California, United Sates).

The experiment was carried out with a portable VR set-up in which participants viewed the virtual environment via an Oculus Rift DK2 (Oculus VR, California, United States) head-mounted display (HMD). The HMD had a resolution of 1920x1080 pixels per eye, updated at 75 Hz with a field of view (FOV) of 100º nominal, 84º horizontal. The set-up included a built-in head tracking device that performed sensor fusion of gyroscope, accelerometer and magnetometer data at 1000 Hz.

A Kinect V2 for Xbox One (Microsoft, Washington, United States) depth sensor was used to enable full-body marker-less tracking. The Kinect's depth sensor had a resolution of 512x424, updated at 30 Hz. The application was developed using the Unity3D 4.5 graphics engine (Unity Technologies, California, United States) and an in-house library was used to integrate in real-time the body tracking from the Kinect with the VR application.

The computer program was executed on an Asus N750JK laptop with an Intel Core i7-4700HQ CPU, with 8GB of RAM and an Nvidia GeForce GTX 850 M graphics card, running Windows 8.1 and DirectX 11. Sound from the virtual environment was played using Sony RF811RK Wireless Headphones (Sony Global, Tokio, Japan).

The emotion recognition test was created and administered with E-Prime 2.0 software (Psychology Software Tools, Pennsylvania, United States) in an Asus N550J laptop with an Intel i7-4700HQ CPU @ 2.40GHz, with 8GB of RAM and an Nvidia GPU 750M graphics card, running Windows 8 (64 bits). The laptop's refresh rate was 60 Hz and the screen resolution was fixed to 1280x720.

Signal Detection Analysis

The *d'* index computes the distance between signal and noise distribution means in standard deviation units. In this study a *d'* of 0 indicates an inability to distinguish angry and fearful facial expressions (signals) from happy facial expressions and body postures since they act as distracters (noise), whereas increasing values of *d'* indicate higher sensitivity, i.e. better skill, to detect angry or fearful facial expressions. The response bias or criterion (*c*) is defined as the distance in standard deviation units between the response criterion and the neutral point where there is no bias towards classifying a face as depicting a certain emotion. A *c* of 0 indicates that participants gave neutral responses without favouring any concrete facial expression, whereas a positive *c* value represents a conservative response (i.e. a bias towards reporting happiness) and a negative *c* value a liberal response (i.e. a bias towards reporting angry or fear, depending on the block of the test).

The *d'* and *c* were calculated based on the following formulas [1,2]:

$$d' = \varphi^{-1}(H') - \varphi^{-1}(F')$$

$$c = -0.5 * [\varphi^{-1}(H') + \varphi^{-1}(F')]$$

where φ^{-1} represents the function that converts probabilities to z scores, H' represents the corrected hit rate and F' the corrected false alarms rate. We used corrected values of $H'$and F' as proposed by Snodgrass & Corwin (3). The concrete formulas used to calculate H' and F' were:

$$H' = (h + 0.5)/(h + m + 1)$$

$$F' = (f + 0.5)/(f + cr + 1)$$

where h (hits) is computed as the proportion of angry and fearful faces correctly classified for male and female stimuli in the corresponding block of the test; f (false alarm) is the proportion of faces that were not expressing either anger or fear and that were incorrectly classified as expressing those emotions; m (misses) is the proportion of angry and fearful faces incorrectly classified as expressing happiness; and cr is the number of correct rejections in noise trials (happy expressions).

We calculated *d'* and *c* indexes for angry and fearful emotion recognition before and after going through the VR experience for female and male stimuli.

Bayesian Analysis

Data analysis was carried out using a Bayesian statistical model following a similar approach as in [4]. It should be noted that we used an overall model where all stochastic equations are treated simultaneously rather than as separate analyses. All prior distributions on the model parameters were chosen to be non-informative (with a very wide variance) and heavily biased against our hypothesis. Through this analysis we obtained the joint posterior distributions of all the model parameters. The measures included in the model were the Social Desirability Scale scores, and the baseline and postIVR minus preIVR difference scores obtained in the Face-Body Compound Test (d' and c indexes). Analysis was carried out using the JAGS system [5], together with MATLAB using MATJAGS[1]. Graphs were produced using Stata 14.

[1] http://psiexp.ss.uci.edu/research/programs_data/jags/

IRI

A validated Spanish version [6] of the Interpersonal Reactivity Index (IRI; 62) was used to measure self-reported dispositional empathy. This questionnaire contains four independent scales that measure affective and cognitive components of empathy. The Perspective Taking (PT) scale measures the reported tendency to spontaneously adopt the psychological point of view of others in everyday life. The Empathic Concern (EC) scale assesses the tendency to experience feelings of sympathy and compassion for unfortunate others. The Personal Distress (PD) scale taps the tendency to experience distress and discomfort in response to extreme distress in others. And the Fantasy (F) scale measures the tendency to imaginatively transpose oneself into fictional situations. Each subscale is addressed by seven items that are answered on a five-point Likert type scale. A summary of the values and differences found in this questionnaire can be seen in **Table S2** and **Fig. S2**.

Table S2. Cohen's d for the difference between Offenders and Controls for the different subscales of the Interpersonal Reactivity Index

Variable Name	Cohen's d for difference between Offenders and Controls[2]
Perspective Taking	0.16
Fantasy	0.26
Empathic Concern	0.40
Personal Distress	0.23

[2] http://rpsychologist.com/d3/cohend/

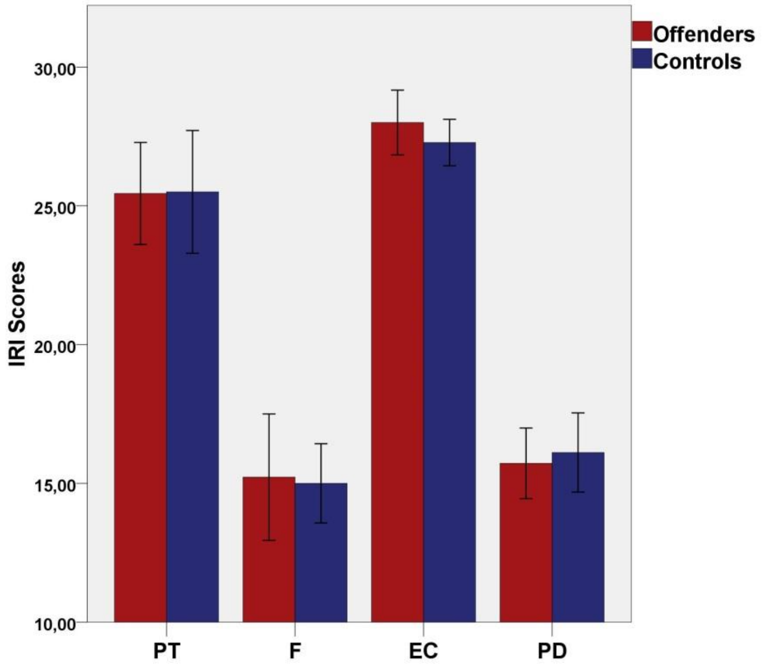

Figure S2. Self-reported empathy traits did not differ between domestic violence male offenders and non-offender male controls. Means and standard errors of the scores obtained in the subscales of the Interpersonal Reactivity Index: Perspective Taking (PT), Fantasy (F), Empathic Concern (EC), and Personal Distress (PD). Higher scores correspond to higher empathic skills.

VR questionnaire

We designed a post VR experience questionnaire to assess participants' subjective perception and attitudes towards different aspects of the VR experience. The questionnaire was divided into five main categories that evaluated different aspects of the virtual scenario: identification with the woman and body ownership, the perception of threat, the interpretation of the scene, responses to the scene, and impact of the scene. Each question (item) was judged on a 1 to 7 Likert-Type Scale, with 1 meaning least and 7 highest agreement with the statement.

- ***Identification with and body ownership of the female virtual avatar***

There is some tendency for Offenders to obtain higher scores on these variables than Controls, but the differences are not particularly strong and might be discounted by the greater propensity of Offenders to obtain higher scores in general due to social desirability (**Table S3**, **Fig. S2**).

Table S3. Effects sizes for the differences between Offenders and Controls in the VR questionnaire responses related to the identification with and body ownership of the female body.

Variable Name	Meaning	Effect Size = proportion of values of Offenders > values of Controls (= total sample size * (Mann-Whitney U statistic))
Identif. with Female	To what extent did you feel identified with the female body during the experience?	0.63
Pers. of Female	To what extent did you put in the perspective of the female avatar and lived the situation as if you were the female?	0.70
Real	To what extent have you experienced the situation as if it was real?	0.66
MyBody	Although you knew it was not your own body, to what extent did you have the illusion that the body of the female avatar was your own body?	0.68

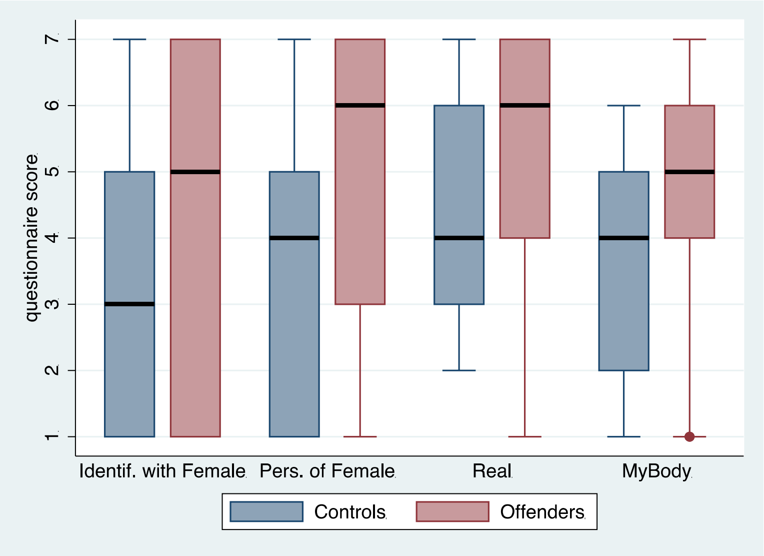

Figure S3. Questionnaire responses related to the identification with and body ownership of the female body.

- ***The perception of threat***

Offenders reported feeling more threatened than Controls, in particular by the shouting and the phone being thrown. Overall, the tendency was for participants to feel threatened (**Table S4**, **Fig. S4**).

Table S4. Effects sizes for the differences between Offenders and Controls in the VR questionnaire responses related to the perception of threat.

Variable Name	Meaning	Effect Size = proportion of values of Offenders > values of Controls (= total sample size * (Mann-

		Whitney U statistic)
ThreatShout	How threatened did you feel when the male avatar shouted?	0.71
ThreatAppro	How threatened did you feel when the male avatar started approaching?	0.64
ThreatGestu	How threatened did you feel when the male was very close and moved his arms?	0.56
ThreatPhone	How threatened did you feel when the male avatar threw the telephone to the floor?	0.71

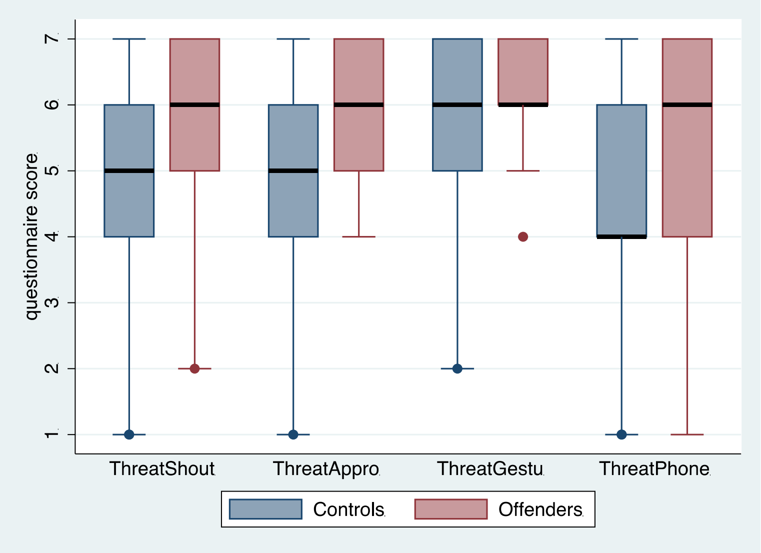

Figure S4. Questionnaire items related to the perception of threat

- ***The interpretation of the scene***

All participants considered the scene as violent, as if they might be physically assaulted and perceived the male character as being abusive, although Offenders considered so to a greater extent (**Table S5**, **Fig. S5**).

Table S5. Effects sizes for the differences between Offenders and Controls in the VR questionnaire items related to the interpretation of the scene.

Variable Name	Meaning	Effect Size = proportion of values of Offenders > values of Controls (= total sample size * (Mann-Whitney U statistic)
ManMirror	Would you have acted differently if the mirror reflection was of a male avatar	0.38

	instead of a female?	
Vulnerable	Did you feel at any time that you were more vulnerable because the character that represented you in the scene was female?	0.48
Violent	How violent do you consider the scene was?	0.78
Assaulted	Did you feel that you could be physically assaulted during the scene?	0.66
AggresAbuse	How aggressive do you consider the abuse of the male avatar was?	0.69

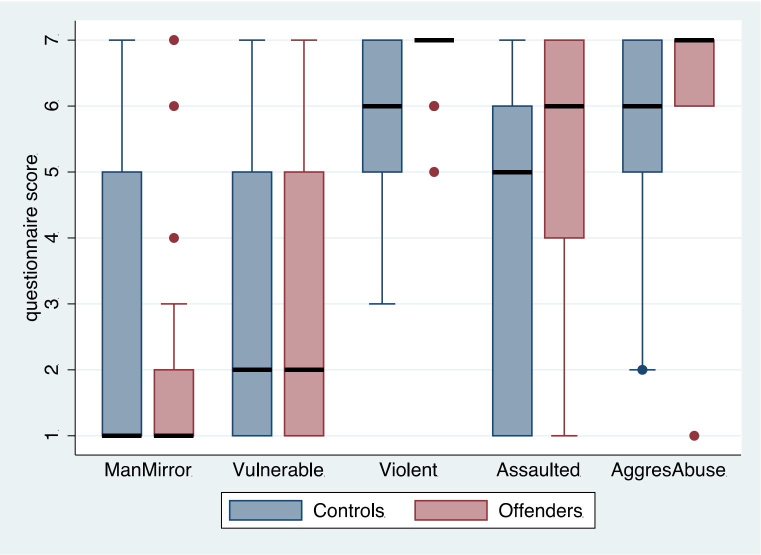

Figure S5. Questionnaire responses related to the interpretation of the scene

- ## Response to the scene

All participants tended to feel that they were being spoken to personally, and wanted to respond although Offenders reported to be less likely to have wanted to physically assault the male character (**Table S6**, **Fig. S6**).

Table S6. Effects sizes for the differences between Offenders and Controls in the VR questionnaire responses related to the responses participants had during the scene.

Variable Name	Meaning	Effect Size = proportion of values of Offenders > values of Controls (= total sample size * (Mann-Whitney U statistic)
Personal	Did you feel that the virtual man was speaking and addressing to you personally?	0.64
Respond	To what extent did you feel like replying back to the male avatar during the scene?	0.54
ToAssault	To what extent did you feel like physically assaulting the male avatar during the scene?	0.34

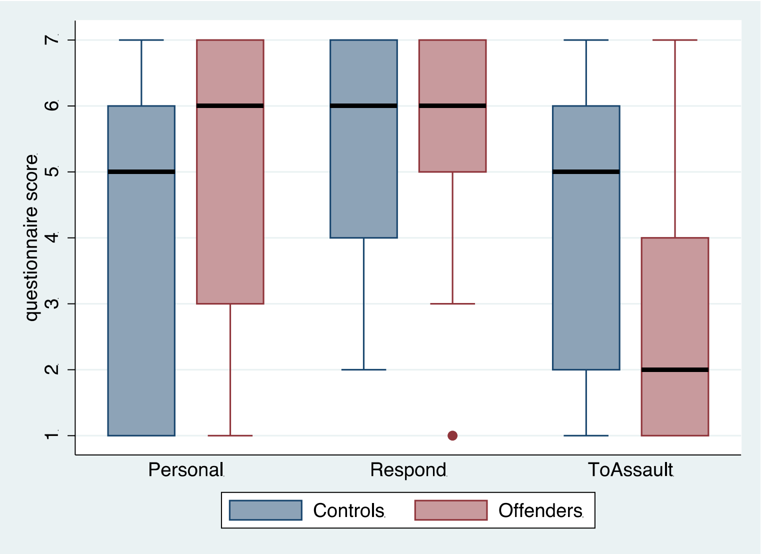

Figure S6. Questionnaire responses related to the responses participants had during the scene.

- ## Impact of the scene

All participants agreed that the experience would be different on a television, and that the virtual scenario helped offenders (or would help offenders, in the case of Controls) empathize and change their behavior with their intimate partner.

Table S7. Effects sizes for the difference between Offenders and Controls in the VR questionnaire items related to the impact of the scene.

Variable Name	Meaning	Effect Size = proportion of values of Offenders > values of Controls (= total sample size * (Mann-Whitney U statistic)
TV	Do you think that the experience would be different if you saw the same scene on a television?	0.38
DiffPersp	Do you think that this experience has allowed you to have a different perspective of this type of situations?	0.48
HelpPersp	Has going through this virtual experience helped you to consider domestic violence from another perspective?	0.78
EmpathyPartner*	To what extent do you think that this scenario can help you to better understand the feelings of your intimate partner? **in case of controls, we ask about how much they think it could help offenders*	0.66
ChangeBeh*	To what extent do you think that this scene can help you change your behaviour with your partner?	0.69

	**in case of controls, we ask about how much they think it could help offenders*	

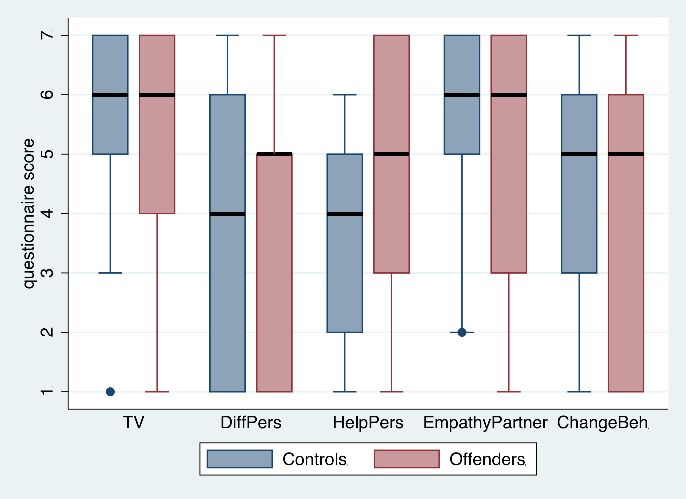

Figure S7. Questionnaire responses related to the impact of the scene.

References

1. Stanislaw, H. & Todorov, N. Calculation of signal detection theory measures. *Behav. Res. Methods, Instruments, Comput.* **31,** 137–149 (1999).

2. Macmillan, N. A. in *A handbook for data analysis in behavioral sciences: Methodological issues* (eds. Keren, G. & Lewis, C.) (Erlbaum, 1993).

3. Snodgrass, J. G. & Corwin, J. Pragmatics of measuring recognition memory: Applications to dementia and amnesia. *J. Exp. Psychol. Gen.* **117,** 34–50 (1988).

4. Bergström, I. *et al.* First-Person Perspective Virtual Body Posture Influences Stress: A Virtual Reality Body Ownership Study. *PLoS One* **11,** e0148060 (2016).

5. Plummer, M. JAGS: A program for analysis of Bayesian graphical models using Gibbs sampling. in *Proceedings of the 3rd international workshop on distributed statistical computing* (Technische Universit at Wien, 2003).

6. Albéniz, A., Paúl, J. de & Etxeberría, J. Adaptación de Interpersonal Reactivity Index (IRI) al español. *Psicothema* **15,** 267–272 (2003).

7. Davis, M. H. Measuring individual differences in empathy: Evidence for a multidimensional approach. *J. Pers. Soc. Psychol.* **44,** 113–126 (1983).
